# Alterations in tidal volume over recording time during pulmonary function testing by barometric whole-body plethysmography in client-owned cats: a multicenter retrospective investigation

**DOI:** 10.1186/s12917-025-04774-0

**Published:** 2025-05-01

**Authors:** Wei-Tao Chang, Laín García-Guasch, Hannah Gareis, Bianka Schulz, Yoshiki Yamaya, Pei-Ying Lo, Chin-Hao Chang, Hui-Wen Chen, Chung-Hui Lin

**Affiliations:** 1 National Taiwan University Veterinary Hospital, National Taiwan University, Taipei, Taiwan; 2 Lab of Small Animal Respiratory and Cardiovascular Medicine, TACS-Alliance Research Center, Taipei, Taiwan; 3 IVC Evidensia Hospital Veterinari Molins & Hospital Veterinaria del Mar, Barcelona, Spain; 4 Veterinary Medicine and Therapeutic Research Group, Faculty of Veterinary Medicine, Research Institute of Biomedical and Health Sciences, University of Las Palmas de Gran Canaria, Las Palmas de Gran Canaria, Spain; 5 Clinic of Small Animal Medicine, Ludwig Maximilian University of Munich, Munich, Germany; 6 Veterinary Anesthesiology & Respiratory Research Laboratory, College of Bioresource Sciences, Nihon University, Kanagawa, Japan; 7 Department of Medical Research, National Taiwan University Hospital, National Taiwan University, Taipei, Taiwan; 8 Department of Veterinary Medicine, National Taiwan University, Taipei, Taiwan; 9 Animal Resource Center, National Taiwan University, Taipei, Taiwan; 10 Graduate Institute of Veterinary Clinical Sciences, School of Veterinary Medicine, National Taiwan University, Taipei, Taiwan

**Keywords:** Lung function test, Tidal breathing, BWBP system, Feline patients, Non-invasive diagnostics

## Abstract

**Background:**

Pulmonary function assessment in small animal clinical patients typically relies on tidal breathing analysis, such as placing cats in a barometric whole-body plethysmography (BWBP) chamber. Despite its wide application in various clinical scenarios, the recording time for BWBP has not been standardized. Variability in resting tidal volume (TV) during natural breathing is significant, impacting the detection of airflow limitation in obstructive airway disease. This multicenter investigation aimed to examine the consistency of TV alterations over the BWBP recording period across different regions, identify influential factors associated with signalment or emotion of the cat, and determine whether the recording time for BWBP in clinical cats could be shorter than in previous studies.

**Results:**

This is a multicenter retrospective study. A total of 131 BWBP recordings from clinical cats across four sites in different countries were enrolled. Ventilatory parameters calculated from short sections using 5 and 20 breaths at the first site showed no significant difference (*P* > 0.05). Among all sites, TV of the initial period (TV-initial, median 32.5 mL, range 4.9-162.9) was significantly greater than TV of the middle (TV-mid, 29.9 mL, range 6.7-144.6) (*P* < 0.001) and the late (TV-late, 27.5 mL, range 5.1-144.8) (*P* < 0.001) period of the recording, while TV-mid and TV-late were not significantly different (*P* = 0.19). The trend of alterations in TV was not affected by site, emotional status, health status, age, or gender. Forest plots with 95% confidence intervals of TV generated from short sections, alongside conventional data averaging breaths over a 5-minute period (TV-All), showed acceptable margins of error at all sites. TV-mid and TV-late were closest to TV-All, whereas TV-initial was larger than TV-mid, TV-late, and TV-All.

**Conclusions:**

TV in clinical cats within the BWBP chamber tends to decrease with recording time. When a shorter section of breaths is desired for clinical evaluation, selecting 5 or 20 breaths from the beginning period of recording would have a higher TV, which might be advantageous for investigating obstructive airway problems. Alternatively, breaths selected from the middle or later stages of recording would be more representative of relaxed breathing over a longer period of time.

## Background

Pulmonary function testing is an essential assessment for obstructive airway disease and various pulmonary disorders, providing objective and quantifiable measures of ventilatory mechanics and aerodynamics [1, 2]. There has been an emphasis on conducting these evaluations in every case with suspicion of chronic obstructive pulmonary disease, and the standard has been continually updated in accordance with the accumulation of knowledge in the field [3–5]. The development of clinical pulmonary function tests in small animal medicine poses greater challenges compared to human patients, primarily because the forced maneuver employed in human medicine requires subject cooperation, which cannot be easily applied to veterinary patients [2, 4, 6]. Consequently, non-invasive pulmonary function assessments in small animal patients are typically limited to tidal breathing analyses [2, 7–9]. This approach mirrors the methodology used in human pediatric or neonatal patients to detect ventilatory functional impairments associated with different respiratory diseases [10–12].

Barometric whole-body plethysmography (BWBP) is a non-restrained system utilized across various animal species to assess tidal breathing parameters, minimizing stress during measurements by allowing animals to move freely within a test chamber [13–18]. Within the BWBP chamber, the animal’s breathing is detected as pseudo-flow, representing the net difference between nasal airflow and thoracic movement [9, 13, 14]. The BWBP method has been proven applicable in client-owned cats, enabling the non-invasive evaluation of pulmonary function parameters in various clinical scenarios and respiratory diseases, such as feline lower airway disease, obesity, and parasitic airway or lung infections [19–26]. Despite its increasing popularity for feline pulmonary function studies, the recording time in various BWBP studies with cats has not been standardized, ranging from 5 to 12 min [15, 19, 20, 26]. Currently, there is no solid consensus on methodological issues in applying BWBP in clinical feline studies.

Technical aspects of pulmonary function testing are also crucial for appropriately interpreting the results, and the associated recommendations should be periodically revised based on collected scientific evidence [4, 5, 12]. The effects of age, body weight, gender, circadian changes throughout the day, the length of acclimatization time, and the methodology to eliminate artifactual waveforms in the obtained BWBP measurements have been reported in cats [15, 27–29]. Nevertheless, many technical issues remain underinvestigated, including the depth of tidal breathing, adequate recording period, and differences among various centers. Considering that resting tidal breathing is highly variable [30, 31], the trend of alterations in tidal volume (TV) during BWBP recording deserves particular attention. In the authors’ observation, TV in some clinical cats tends to progressively decrease as the recording time lengthens [32], presumably because the animals become more accustomed and relaxed within the chamber. This reduction in TV, in contrast to maximal respiratory effort, may lead to undetected airflow limitation in obstructive airway disease owing to insufficient respiratory effort [33].

The objectives of this study were to assess alterations in TV over the BWBP recording period, investigate the consistency of the trend in different regions, evaluate whether the recording time for BWBP in clinical cats could be shorter than previous studies, and examine factors associated with emotional status, sex, and age.

## Methods

This multicenter retrospective study included tidal breathing data recorded using BWBP in client-owned cats, recruited from veterinary medical centers across four countries. At each site, investigators selected a minimum of 10 and a maximum of 40 clinical cases that underwent BWBP recording for various clinical reasons from their pulmonary function database. Ventilatory parameters derived from the tidal breathing of multicenter origin were integrated for analysis. Information regarding the breed, age, gender, body weight, health status, site, operator of BWBP, and the emotional status (calm or nervous, as categorized by the operator) of the cat during recording were retrospectively collected.

The BWBP recording performed at each site was based on the manufacturer’s instructions (Buxco Electronics and Data Science International). The primary components of the system consist of a transparent animal chamber, bias flow regulator, differential pressure transducer, analog-to-digit converter, and software, which were similar among sites. The calibration process was performed according to the instructions for each version of the system (site A: Buxco hardware with BioSystem XA 2.11.0 software; site B: Buxco hardware with Biosystem XA 2.10.1 software; site C: Buxco/DSI hardware with FinePoint 2.4.6.9414 software; site D: Buxco/DSI hardware with FinePoint 2.3.19 software). The cat was usually allowed to acclimatize to the chamber for a period of time before recording. The acclimation protocol varied among sites, either using a fixed period or based on the individual status of each cat. An ordinary acclimation period ranged from 1 to 10 min, while a prolonged acclimation protocol lasted 20 min. Conventional BWBP parameters calculated by the software included respiratory rate (RR; breaths/min), tidal volume (TV; mL), minute volume (MV; mL), inspiratory and expiratory times (Ti and Te; s), peak inspiratory and expiratory flow (PIF and PEF; mL/s), relaxation time (RT; s; time point when 65% of tidal volume is expired), pause (unitless; [Te– RT]/ RT), and enhanced pause (Penh; unitless; [PEF/PIF] x pause).

The breathing parameters from the BWBP system at each site were exported in Excel format. Variables related to rate (RR), volume (TV), flow rate (PIF and PEF), and a composite index from multiple parameters (Penh) were used as representative data for each recording. For each case, the initial and late periods referred to the first and last few breaths of the recording, respectively, while the middle period was defined as the breaths occurring around the midpoint between the initial and late periods. For sites that only recorded data after acclimation, the first few breaths in the exported file were used to represent the initial period (sites A, B, and D). Alternatively, for a site that included data from a prolonged acclimation period in the exported file (site C), the first 5 min of data were excluded to simulate the acclimation period used at other sites. Data from breaths recorded over a 5-minute period were averaged and referred as ‘conventional data’ (TV-All). Ventilatory parameters from three short sections at the initial, middle, and late periods of the recording were calculated using 5 and 20 breaths, respectively, at site A to determine the number of breaths for subsequent analyses. For the multicenter analysis of alterations in TV over recording time, 20 breaths from the initial (TV-initial), middle (TV-mid), and late (TV-late) periods of the recording were averaged and used for sites that exported breath-by-breath data (site A, B, and D). Alternatively, average values calculated from 30-second data of the initial, middle, and late periods were used for a site that exported mean breath values every ten seconds instead of breath-by-breath data due to its system settings (site C).

### Statistical analysis

Statistical analyses were conducted using SPSS version 26 (IBM Corp, USA). Normal distribution was assessed using the Shapiro-Wilk test. Data with a normal distribution were presented as mean and standard deviation (SD), while non-normally distributed data were presented as median and range. The baseline signalment of the four sites was compared using the Kruskal–Wallis test. When significance was demonstrated, a post-hoc Bonferroni correction was applied. Alterations in TV calculated from different numbers of breaths or at different stages of recording, were compared using the Wilcoxon signed-rank test. Generalized estimated equation (GEE) analysis with an autoregressive structure was applied to evaluate whether factors such as sites, emotional status of the cat, health status of the cat, gender, and age could potentially affect the trend of changes in TV over time, assessing the interaction effect of the factors and time on TV. A *P* value of less than 0.05 was considered statistically significant.

## Results

A total of 131 recordings from clinical cases, including 11 healthy cats free of respiratory signs and 120 cats with various respiratory diseases, underwent pulmonary function testing using BWBP for various reasons across four sites (Site A: 40 cats; Site B: 40; Site C: 40; Site D: 11) and were enrolled in the analysis. The median age of all study cats was 7.0 years (range 0.6–17.0 years) with a median body weight of 4.5 kg (range 2.5–10.6 kg). There was no significant difference in age among the four different sites, but cats at site A presented with a higher median body weight compared to those at site B (4.8 kg, range 3.2–10.6 versus 4.0, range 3.0-7.8, *P* = 0.01).

**Fig. 1:**
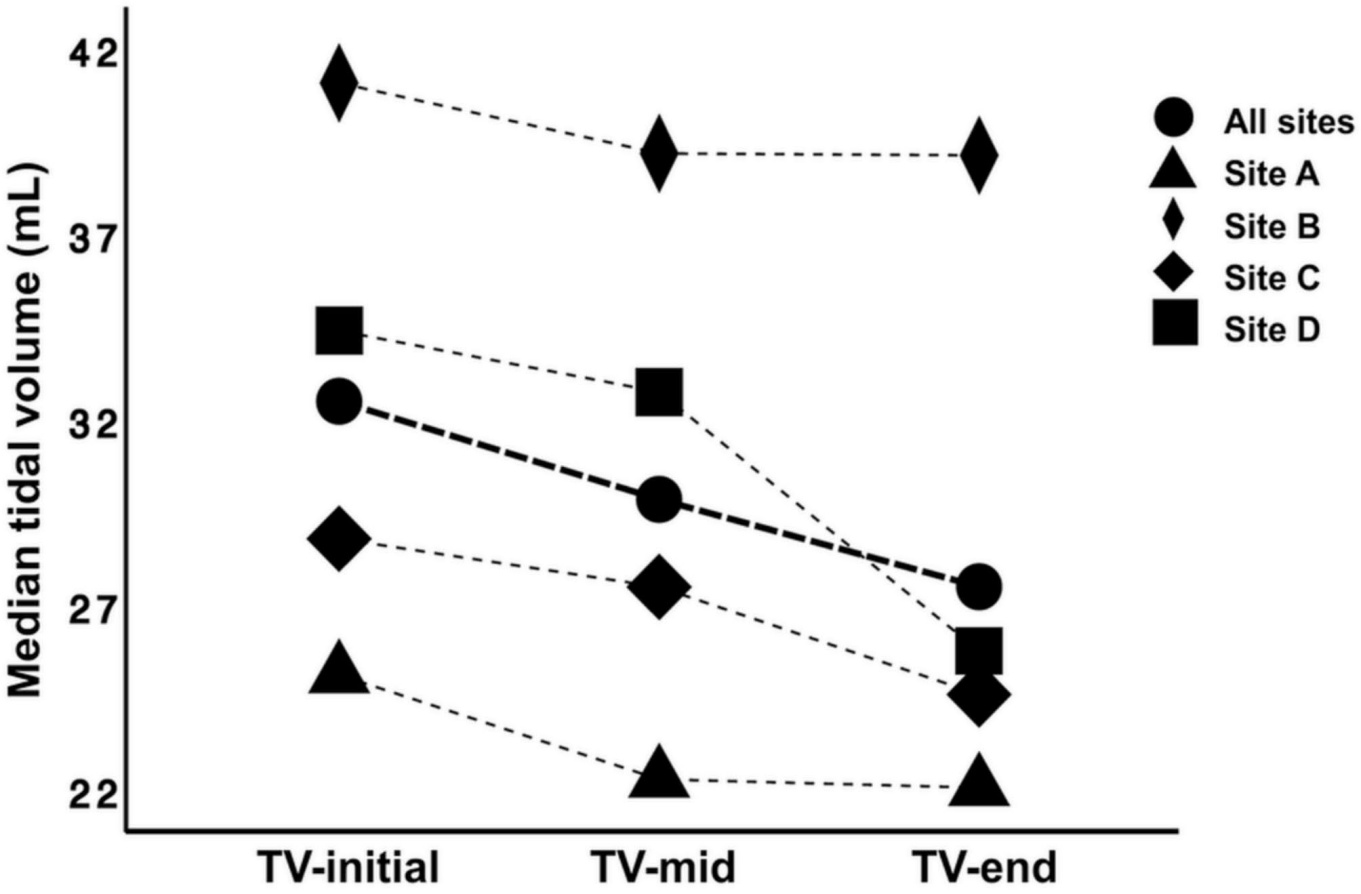
Alterations of tidal volume (TV, mL) were visually depicted for the initial, middle, and late periods of the recording, demonstrating the trend over recording time. The solid circle and thick line represent the overall trend of all sites, while the trend of individual sites is illustrated with different symbols and thin lines. (Abbreviations: TV-initial, TV-mid, and TV-late represent tidal volume of the initial, middle, and the late period of the recording)

Ventilatory parameters were calculated from three sections (initial, middle, and late periods of the recording) at site A using 5 and 20 breaths, respectively. The initial and late periods referred to the first and last 5 or 20 breaths of the recording, respectively, while the middle period used breaths around the midpoint of the recording. No significant difference was found between the datasets of 5 and 20 breaths (*P* > 0.05); thus, data from 20 breaths were selected by the investigators for subsequent multicenter analysis. At one site, where breath-by-breath data was unavailable and the system exported mean values every ten seconds, 30-second data were used to match the data length calculated from 20 breaths at other sites. The variability of TV appeared substantial across individual sites, as cases were selected casually, without restriction to a specific disease category, to enhance the generalizability of the investigation into TV alterations during recording. For the analysis of alterations in TV over recording time, TV of the initial period (TV-initial, median 32.5 mL, range 4.9-162.9) was significantly greater than TV of the middle (TV-mid, median 29.9 mL, range 6.7-144.6) (*P* < 0.001) and the late (TV-late, median 27.5 mL, range 5.1-144.8) (*P* < 0.001) period of the recording, while TV-mid and TV-late were not significantly different (*P* = 0.19) (Fig. 1). Meanwhile, the respiratory rate during the late period (49 breaths/min, range 14–216) was also significantly lower than that of the initial period (60 breaths/min, range 15–251) (*P* = 0.003), representing a more profound resting state of cats after prolonged staying in the box.

In the GEE analyses investigating potential factors influencing the trend of alterations in TV over time, this phenomenon was not found to be significantly affected by sites, emotional status (calm versus nervous), health status (healthy versus diseased), age, or gender (Table 1).

**Table 1:** Generalized estimating equations for the influence of sites, emotional status (calm versus nervous), health status (healthy versus diseased), age, gender on the trend of the decrease in TV over time

Variables	Beta coefficient	95% CI	X^2^	df	*P* value
Time	-2.168	(-3.204, -1.132)	16.834	1	< 0.001
Time x Site A					
Time x Site B Time x Site C Time x Site D	0.428 -1.477 -0.172	(-2.441, 3.296) (-3.413, 0.459) (-2.511, 2.166)	0.085 2.235 0.021	1 1 1	0.77 0.14 0.89
Time x Calm					
Time x Nervous	-0.935	(-3.249, 1.378)	0.628	1	0.43
Time x Diseased					
Time x Healthy	-2.846	(-6.078, 0.386)	2.980	1	0.08
Time x Female					
Time x Male	-1.32	(-3.497, 0.637)	1.839	1	0.18
Time x Age	0.117	(-0.083, 0.318)	1.312	1	0.25

CI, confidence interval; df, degree of freedom; X^2^, chi square statistic

**Fig. 2:**
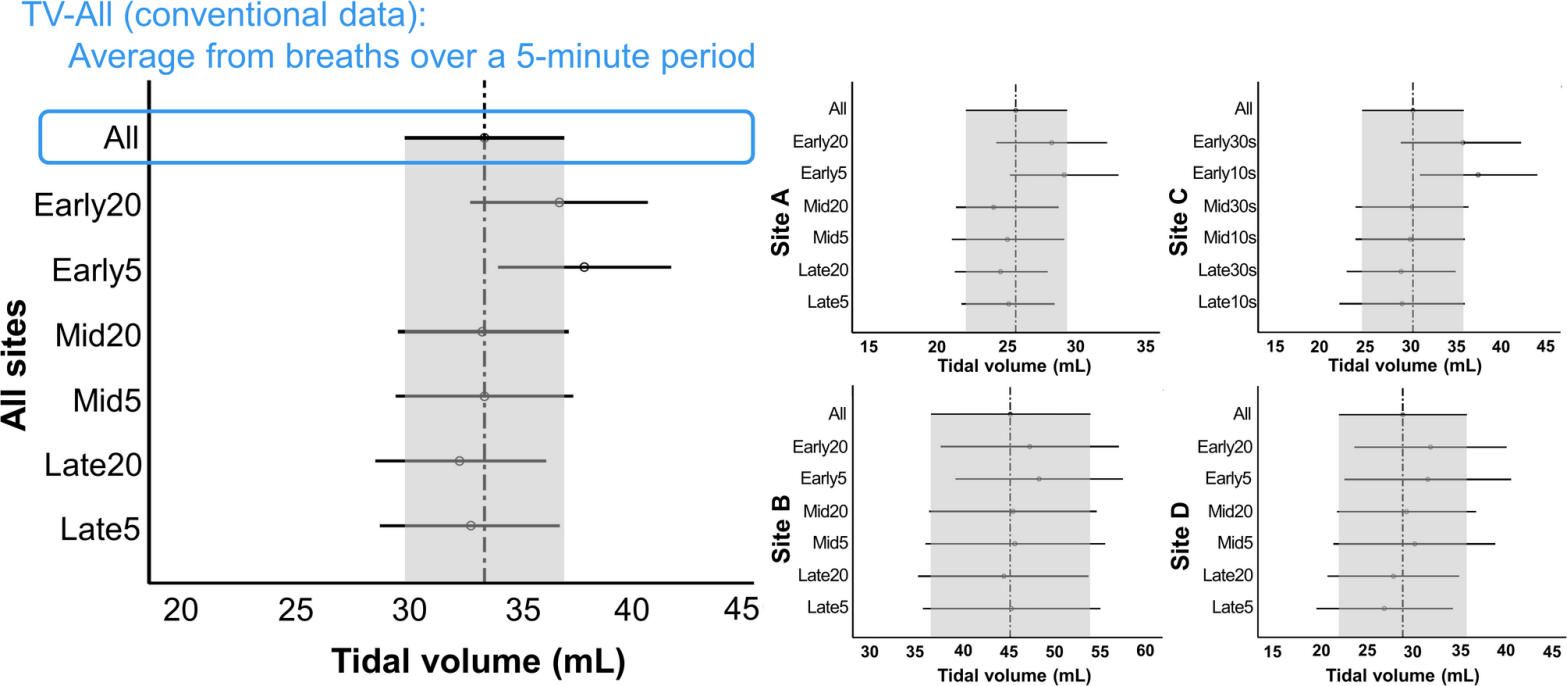
The forest plots showed the 95% confidence intervals (CI) of six datasets of tidal volume (TV), obtained from short sections comprising several breaths from the initial, middle, or late periods of the recording. The plots depicted data from all sites (left) and 4 individual sites (right). At the top of each forest plot, the conventional data averaging breaths over a 5-minute period (TV-All) was displayed. The vertical line and gray area represented the median and the 95% CI range of TV-All, respectively. (Abbreviations: Initial20, Mid20, and Late20 represent data calculated from 20 breaths of the initial, middle, and late period of the recording; Initial5, Mid5, and Late5 represent data from 5 breaths of each period; Initial30s, Mid30s, and Late30s represent data from 30 s of recording of the initial, middle, and late period; Initial10s, Mid10s, and Late10s represent data from the average of 10 s recording of each period)

The forest plot was used to illustrate the 95% confidence intervals (CI) of six TV datasets, each representing different sections (breaths from the initial, middle, or late periods of the recording), alongside the conventional data averaging breaths over a 5-minute period (TV-All) (Fig. 2). The overall margins of error were acceptable for all six datasets, both for the integrated data of all sites and for each individual site. The calculation of TV from the middle or late period of the recording was closest to TV-All, whereas TV derived from the initial stage of recording was larger than TV-All or data from the middle or late period.

## Discussion

This multicenter investigation examined changes in TV over recording time and explored technical factors that could influence the outcome variables during pulmonary function testing using the BWBP method in client-owned cats. Our data demonstrated a consistent trend across different sites, with TV gradually decreasing within minutes, unaffected by the cats’ emotional or health status, gender, or age. Regardless of whether they initially appeared nervous or calm, cats settled into a more relaxed state, as also supported by a lower respiratory rate in the later stage of the recording, indicating a more restful state. These results suggest that clinical cats tend to exhibit a higher TV when first introduced into the BWBP chamber, followed by a decrease in TV within a few minutes as they become more accustomed to the environment.

The current methodology for assessing pulmonary function differs significantly between small animals and human clinical patients. The standard practice in human medicine involves applying forced maneuvers to measure the maximal volume of air expired and inspired with maximal effort, as forcefully and completely as possible [4]. The primary rationale is that maximizing the magnitude of expiratory driving pressure could help reveal pathological airflow limitations associated with obstructive ventilatory impairments [5, 34]. On the contrary, relaxed tidal breathing utilizes only a small fraction of ventilatory capacity, causing its sensitivity in detecting small airway obstruction to vary among cases; it proves effective in certain instances while proving inadequate in others [12]. Furthermore, tidal breathing can be highly variable and complicated, as the driving pressure from expiratory muscle activity could be affected by multiple factors, including disease factors or conscious effort [12, 30]. Although recording tidal breathing in cats with BWBP method is relatively simple, interpreting it can be challenging, and technical aspects remain underinvestigated in the field of veterinary medicine.

Nevertheless, expiratory flow patterns during tidal breathing can still reveal distinguishable features in patients with various respiratory diseases compared to normal individuals [10–12]. It has been reported that the patterns of tidal breathing flow-volume loops in infants and young children with peripheral bronchoconstriction, airway obstruction, and chronic lung disease differ from those of healthy controls [10, 11]. While interpretation of results derived from tidal breathing measurements should be approached with caution due to its complexity, it is considered a noninvasive and clinically useful method providing insightful pathophysiological information in infants and some preschool children [10, 12]. Tidal breathing analysis also showed differences between cats and dogs with respiratory diseases and normal animals, such as obstructive airway disease, tobacco exposure, obesity, and parasite-associated lung disease [7, 8, 17, 18, 20–26]. Despite not being as sensitive and reliable as a forced maneuver, analysis of tidal breathing remains an applicable method for patients unable to consciously apply expiratory effort as instructed.

Our results demonstrated that TV tends to decrease with the length of recording time as cats become more accustomed and relaxed in the BWBP chamber. However, a smaller TV is not desirable in many clinical evaluations because it hinders the ability to detect abnormalities, such as in lung sound auscultation. A small TV has also been recognized as a potential limitation for pulmonary function tests. The smaller the expiratory volume from forced expiration, the less likely it is to reach the flow limitation necessary for detecting airway obstruction in breathing mechanics [6, 10, 33]. Consequently, multiple techniques that artificially enhance ventilatory efforts have been adopted to increase TV during pulmonary function tests, such as breathing CO2 or administering doxapram [33, 35, 36]. In future assessments aimed at detecting obstructive problems, selecting breaths from the initial rather than middle or later stages might increase the likelihood of detecting abnormalities by maximizing TV. On the other hand, if the purpose of an examination is to assess the stable ventilatory status, breaths from the middle or later stages of recording would be more representative of relaxed breathing over a period of time.

The trend of alterations in TV over recording time in cats appears to be a global phenomenon with some minor variations among different sites, unrelated to the emotional status, health status, gender, and age of the cats included in the study. While emotional status was expected to influence TV and its change over time, an acclimation period allowing cats to become accustomed to the environment, along with the subjective judgment of clinicians discerning nervousness from calmness, can mask the effects of emotion. A recent study demonstrated that different acclimation times significantly affected ventilatory parameters in healthy cats; however, TV did not change significantly among the three time periods over a 30-minute recording in healthy cats [29]. In that study, each period consisted of 10 min of recording for a total of 30 min. However, in our multicenter analysis with mostly diseased cats, only 20 breaths, occurring approximately within 30 s (sites A, B, D), or breaths calculated from 30-second data (site C), were analyzed. This was because our study also aimed to evaluate whether the recording time for clinical cats could be shorter than in previous studies. Although the two studies might not be directly comparable, together they indicate that technical aspects such as the definition of acclimatization or the recording period are crucial factors that should be selected according to clinical purpose and addressed in future studies of pulmonary function testing.

Potential limitations of the current study must be considered. Firstly, both healthy and diseased cats were included in this study with the purpose of reflecting clinical reality across different sites; however, the differences between health and disease in ventilation among conscious cats remain unclear. Secondly, the hardware and software of the BWBP systems vary among study sites. Although it is unknown whether the factors associated with the device would influence the present data, such variability is inevitable in any multicenter investigation involving medical or specialized research equipment. Thirdly, the method of acclimation, data acquisition using breath-by-breath or average-per-period methods, and the criteria for assigning emotional statuses to cats were not standardized, as this study was designed to retrieve recordings from the pulmonary function databases of each center. Despite these being common flaws inherent to retrospective studies, the findings of our study emphasize the importance of technical factors and encourage prospective investigations in the future.

## Conclusion

In conclusion, TV in clinical cats within the BWBP chamber tends to decrease with the length of recording time in this multicenter investigation. The alteration in TV was similar across different sites, and the phenomenon was not statistically influenced by emotional states, gender, or age of the cats. When a shorter period of breaths is desired for clinical evaluation (e.g., in cases where CO2 breathing or doxapram administration is needed), selecting 5 or 20 breaths shortly after the cat’s initial introduction into the BWBP chamber would result in a higher TV, which might be advantageous for investigating obstructive airway problems. Alternatively, selecting 5 or 20 breaths from the middle or later stages of the recording would better represent relaxed breathing over a prolonged recording period.

## Data Availability

The datasets used and/or analysed during the current study are available from the corresponding author on reasonable request.

## Abbreviations

BWBP: Barometric whole-body plethysmography
CI: Confidence intervals
GEE: Generalized estimating equation
MV: Minute volume
PEF: Peak expiratory flow
Penh: Enhanced pause
PIF: Peak inspiratory flow
RR: Respiratory rate
RT: Relaxation time
SD: Standard deviation
Te: Expiratory time
Ti: Inspiratory time
TV: Tidal volume
